# Foley Follies: Emphysematous Pyelitis from Instrumentation in Obstructive Uropathy

**DOI:** 10.7759/cureus.1612

**Published:** 2017-08-26

**Authors:** Shimshon Wiesel, Anna Gutman, Jonah E Abraham, Militza Kiroycheva

**Affiliations:** 1 Internal Medicine, Staten Island University Hospital, Northwell Health; 2 Department of Anesthesiology, University of Pittsburg Medical Center; 3 Department of Nephrology, Staten Island University Hospital, Northwell Health

**Keywords:** urinary tract infection, acute kidney injury, emphysematous pyelitis, foley catheter, iatrogenic injuries, hydronephrosis

## Abstract

Emphysematous pyelitis (EP) is a subclass of a life-threatening necrotizing infection of the urinary system called emphysematous pyelonephritis (EPN). We report a case of an 81-year-old man with emphysematous pyelitis, which occurred after urinary tract instrumentation and resolved with conservative medical management. This case highlights the potential complications of urinary tract manipulation and the importance of a prompt diagnosis.

## Introduction

Emphysematous pyelitis (EP) is a subclass of a necrotizing infection of the urinary system called emphysematous pyelonephritis (EPN). EPN is a life-threatening medical emergency occurring mostly in women with diabetes mellitus (DM). We report a case of an 81-year-old nondiabetic man who developed EP after urinary tract manipulation.

## Case presentation

An 81-year-old man presented to our hospital for infected lower extremity ulcers and worsening kidney function. His past medical history was significant for benign prostatic hyperplasia, chronic kidney disease stage IIIb, hypertension, and peripheral artery disease. On physical examination, he had a temperature of 98.7F, heart rate of 85 beats per minute, respiratory rate of 16 breaths per minute, blood pressure of 138/72 mm Hg, and oxygen saturation of 98% when breathing ambient air. The patient had a 1+ pitting edema in both lower extremities and infected ulcers on the right hallux and the right calf. The rest of his physical examination was normal.

The patient's serum creatinine (sCr) was 3.12 milligrams per deciliter (mg/dL), which was elevated from his outpatient baseline of 1.69 mg/dL, and urea nitrogen was 32 mg/dL. His urinalysis showed small blood, large leukocytes, and 30 mg/dL of protein on urine dipstick, 20 to 30 white blood cells per high power field (p/hpf), three to six red blood cells p/hpf, few hyaline casts p/hpf, and occasional mucous threads on microscopic examination. His serum total protein, albumin, complement, low-density lipoprotein, and high-density lipoprotein levels were normal. Antiproteinase 3 antibodies, anti-myeloperoxidase antibodies, and antinuclear antibodies were negative. A renal ultrasound showed bilateral hydroureteronephrosis and a postvoid residual urine volume of 350 milliliters.

**Figure 1:**
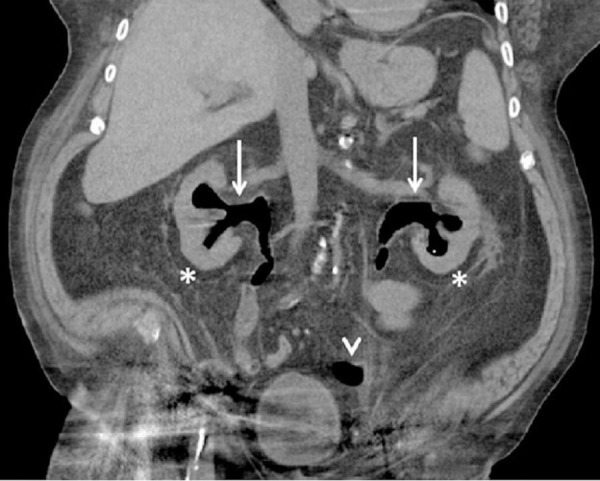
Coronal CT abdomen and pelvis A coronal view illustrating air in the bilateral renal pelvises with extension into the ureters (arrows) without parenchymal renal involvement. The fat-stranding of the bilateral kidneys is illustrated (asterisks). There is also air at the left ureterovesicular junction (arrowhead). CT = computed tomography

An indwelling urinary catheter was placed, and intravenous fluids were administered. The patient’s sCr level improved 24 hours after introducing the urinary catheter. A repeat ultrasound was done 48 hours after the urinary catheter placement, which showed bilateral renal collecting shadowing echogenic foci, consistent with possible EP. A computed tomography (CT) scan of the abdomen and pelvis without contrast showed mild bilateral hydronephrosis with air seen throughout the collecting systems and ureters (Figure 1). The urine gram stain and culture were negative and the patient’s antibiotic regimen was changed from ampicillin-sulbactam to vancomycin and ertapenem empirically due to a recent hospitalization and antibiotics use.

Throughout his hospitalization, the patient denied abdominal pain, flank pain, suprapubic pain, dysuria, or urinary urgency, and he remained afebrile, with stable vital signs. His renal function improved to its pre-admission baseline, so a percutaneous catheter drainage (PCD) of the renal pelvises was not performed. The patient was sent home with the urinary catheter in place on an antibiotics regimen of vancomycin and ertapenem for a total of 14 days. He was instructed to have a close follow-up with his primary care physician, urologist, and nephrologist. Follow-up details were obtained from the patient’s primary care physician, who informed us that a subsequent CT of the patient’s abdomen and pelvis demonstrated complete resolution of his EP. 

## Discussion

EPN is a rare, but life-threatening, acute necrotizing infection of the kidney, first clinically described by Kelly and MacCallum in 1898. EPN is defined as gas within the renal parenchyma, collecting system, or perinephric tissues [1]. EP is a subclass of EPN, in which gas is confined to the renal collecting system.

EPN is a disease more commonly seen in women than in men, with a female-to-male ratio of 3 to 1, presumably because of women's increased susceptibility to urinary tract infections [1]. Over 90% of patients with EPN have diabetes mellitus (DM) [2], whereas EP is usually associated with urinary tract obstruction [3]. Only 50% of patients with EP have DM, compared to the > 90% of patients with EPN class II or higher. The most common cause of urinary tract obstruction that leads to EP is renal calculi [4].

Urinary tract obstruction causes EPN by impairing the transportation of formed gas, which increases local pressure in the tissue and impairs circulation. Impaired circulation leads to infarction, which provides a culture medium for gas-forming pathogens, thereby creating a vicious cycle [3]. Gas appearing in the urinary system may also be caused by fistulae originating in the gastrointestinal tract, gas reflux from the urinary bladder, trauma, and urinary system interventional procedures [4].

EPN is caused by organisms that ferment glucose and lactate into carbon dioxide and hydrogen gas. The organisms involved are usually enteric facultative anaerobic bacteria, such as Escherichia coli, Proteus mirabilis, Klebsiella pneumoniae, Enterococcus species, and Pseudomonas aeruginosa; occasionally, gram positive bacteria, such asStreptococci species; and fungi such as Candida albicans[1]. Patients with DM provide a favorable environment for gas-forming microbes because of the high levels of glucose in their tissues and urine, which is used as a substrate for fermentation. 

The common clinical features of EPN include fever, chills, costovertebral angle tenderness, vomiting, dysuria, and, rarely, crepitus in the lumbar region [1]. The risk factors associated with high mortality rates from EPN are thrombocytopenia, altered mental status, severe proteinuria, shock, extension of the infection to the perinephric space, renal replacement therapy dependence, hypoalbuminemia, and polymicrobial infections [1,5-6].

The diagnosis of EPN is established by demonstrating gas in the urinary system, renal parenchyma, or perinephric tissue by a plain abdominal radiograph or a renal ultrasound. A CT scan of the abdomen and pelvis can confirm the diagnosis and show the extent of the disease, thus being the ideal test for the diagnosis of EPN [5]. A poor response to antibiotic therapy in a patient with DM, with a seemingly uncomplicated pyelonephritis, should raise suspicion of EPN and warrant a CT scan of the abdomen and pelvis to rule out this life-threatening condition [1].

EPN is divided into four classes [5], which are determined according to the extent of the gas expansion (Table 1). The gas may be limited to the collecting system or expand through the renal parenchyma and retroperitoneal space. Miller et al. described a severe case of EPN, in which gas extended through the retroperitoneal space and invaded the inferior vena cava [2].

The treatment of EPN remains controversial. Early nephrectomy was the treatment of choice in the 1980s, with a general consensus that medical therapy without surgical intervention has higher rates of mortality [7]. Studies in the 1990s proposed antibiotic therapy combined with CT-guided percutaneous drainage (PCD) as an acceptable alternative to nephrectomy [8], which lowered mortality rates by as much as 21% [6]. Further studies in the last two decades continue to demonstrate a significantly lower mortality rate with PCD alongside medical management compared to early nephrectomy in most classes of EPN [6,8].

Empiric antimicrobials should primarily target gram-negative bacteria. Third-generation cephalosporins are recommended as the initial treatment of EPN. Carbapenems, frequently used in combination with vancomycin, are the empiric antibiotic of choice for patients with prior hospitalization and antibiotics use within the last year, patients requiring hemodialysis, and patients who develop disseminated intravascular coagulation. PCD and the placement of double-J catheters are often performed in conjunction with medical treatment to maximize nephron sparing, which significantly lowers the rate of mortality. Salvage nephrectomy or open drainage is usually performed when PCD or conservative treatments fail or have a high probability of failing [5,9]. A 25% mortality rate has been observed in patients undergoing an emergency nephrectomy, 50% with medical management, and only 13% with medical management and PCD [10].

Huang and Tseng proposed common guidelines for the treatment of EPN (Table 1). Most classes of EPN require antibiotics and PCD. Nephrectomy is currently reserved patients with class III EPN with multiple risk factors and class IV if antibiotics and PCD fail.

**Table 1:** EPN classification and management ^*^For all classes: relief of any existing urinary tract obstruction ^**^Emphysematous pyelitis ***That is, thrombocytopenia, acute kidney injury, altered mental status, shock PCD = Percutaneous drainage

Class	Gas Location	Management^*^
I^**^	collecting system	antibiotics and PCD
II	renal parenchyma	antibiotics and PCD
IIIA	extension to the space between the fibrous renal capsule and the renal fascia (perinephric space)	< 2 risk factors^***^, PCD and antibiotics; 2 or more risk factors, early nephrectomy
IIIB	extension to the space beyond the renal fascia and/or extension to adjacent tissues (pararenal space)	< 2 risk factors^***^, PCD and antibiotics; 2 or more risk factors, early nephrectomy
IV	bilateral kidneys or a solitary functioning kidney	trial of antibiotics and PCD; nephrectomy if antibiotics and PCD fail

## Conclusions

Our patient had a negative urine culture, however, his concomitant antibiotic coverage for a coexisting infection may have altered the validity of the urine cultures. Nevertheless, the differential diagnosis of air in the collecting ducts of the urinary system is not confined to infectious causes and the placement of a urinary catheter in the setting of a postrenal obstruction may have caused an iatrogenic EP. Individual analyses of the risks and benefits of choosing surgical intervention in addition to medical management should be evaluated on a case-by-case basis.
